# Adult Medulloblastoma: Updates on Current Management and Future Perspectives

**DOI:** 10.3390/cancers14153708

**Published:** 2022-07-29

**Authors:** Enrico Franceschi, Caterina Giannini, Julia Furtner, Kristian W. Pajtler, Sofia Asioli, Raphael Guzman, Clemens Seidel, Lidia Gatto, Peter Hau

**Affiliations:** 1 Nervous System Medical Oncology Department, IRCCS Istituto delle Scienze Neurologiche di Bologna, Via Altura 3, 40139 Bologna, Italy; 2 Department of Laboratory Medicine and Pathology, Mayo Clinic, Rochester, MN 59005, USA; giannini.caterina@mayo.edu; 3 Department of Biomedical and Neuromotor Sciences (DIBINEM), University of Bologna, 40126 Bologna, Italy; sofia.asioli@ausl.bologna.it; 4 Department of Biomedical Imaging and Image-Guided Therapy, Medical University of Vienna, 1090 Vienna, Austria; julia.furtner@meduniwien.ac.at; 5 Hopp Children’s Cancer Center Heidelberg (KiTZ), 69120 Heidelberg, Germany; k.pajtler@kitz-heidelberg.de; 6 Division of Pediatric Neurooncology, German Cancer Research Center (DKFZ) and German Consortium for Translational Cancer Research (DKTK), 69120 Heidelberg, Germany; 7 Department of Pediatric Oncology, Hematology and Immunology, Heidelberg University Hospital, 69120 Heidelberg, Germany; 8 Pituitary Unit, IRCCS Istituto Delle Scienze Neurologiche Di Bologna, Via Altura 3, 40139 Bologna, Italy; 9 Department of Neurosurgery, University Hospital of Basel, 4031 Basel, Switzerland; raphael.guzman@usb.ch; 10 Department of Radiation Oncology, University Hospital Leipzig, 04103 Leipzig, Germany; clemens.seidel@medizin.uni-leipzig.de; 11 Department of Oncology, AUSL of Bologna, 40139 Bologna, Italy; lidia.gatto@ausl.bologna.it; 12 Wilhelm Sander NeuroOncology Unit & Department of Neurology, University Hospital Regensburg, 93055 Regensburg, Germany; peter.hau@ukr.de

**Keywords:** medulloblastoma, neurosurgery, radiotherapy, chemotherapy, targeted therapy, sonic hedgehog (SHH), vismodegib, sonidegib, SHH pathway, SHH inhibitors

## Abstract

**Simple Summary:**

Adult medulloblastoma is an extremely rare tumor of the central nervous system. Standard multimodal treatment, comprising maximal safe surgical resection followed by craniospinal radiotherapy and multi-agent chemotherapy, can improve the prognosis of this disease, producing, however, important acute and long-term toxicities. Herein, we review the state of the art for adult medulloblastoma diagnosis and treatment, presenting novel molecular advances and their therapeutic implications and discussing the central role of hub centers to guarantee the highest quality of care and a better overall outcome for this rare tumor.

**Abstract:**

Medulloblastoma (MB) is a malignant embryonal tumor of the posterior fossa belonging to the family of primitive neuro-ectodermic tumors (PNET). MB generally occurs in pediatric age, but in 14–30% of cases, it affects the adults, mostly below the age of 40, with an incidence of 0.6 per million per year, representing about 0.4–1% of tumors of the nervous system in adults. Unlike pediatric MB, robust prospective trials are scarce for the post-puberal population, due to the low incidence of MB in adolescent and young adults. Thus, current MB treatments for older patients are largely extrapolated from the pediatric experience, but the transferability and applicability of these paradigms to adults remain an open question. Adult MB is distinct from MB in children from a molecular and clinical perspective. Here, we review the management of adult MB, reporting the recent published literature focusing on the effectiveness of upfront chemotherapy, the development of targeted therapies, and the potential role of a reduced dose of radiotherapy in treating this disease.

## 1. Introduction

First described by Bailey and Cushing in 1925 [1], MB is a rare embryonic tumor of the posterior cranial fossa, grade 4 assigned by WHO classification, arising from the granule cell precursors in the external germinal layer of the developing cerebellum.

Tumor growth starts in the fourth ventricle and has a propensity to spread to the cerebellar vermis and the brainstem, seeding the craniospinal axis through the subarachnoid system (the so-called “drop metastases”) [2,3].

Examining data from the Surveillance, Epidemiology, and End-Results (SEER) database from 1973 through 2007, MB is the second most common of all pediatric central nervous system (CNS) tumors (accounting for about 20% of the total CNS tumors in the pediatric age group), with a bimodal peak at age 3 to 4 years and at age 8 to 10 years [3,4]. Age of MB onset is variable, from birth to adolescence, but the incidence rate dramatically declines after age 15 [5].

In adult neuro-oncology practice, therefore, MB is a very rare condition, representing only 0.4–1% of all adult CNS tumors, with an incidence of 0.6–1 case per million per year [4]: this population, which is the topic of this review and for readability purposes will be called “adult MB”, is an “orphan” entity, being that the main therapeutic protocols were extrapolated from experiences in children, while prospective randomized clinical trials specifically designed for adult patients are very rare [5,6,7,8,9,10].

Current standard treatment involves a multimodal treatment comprising surgical resection followed by radio-chemotherapy, selected and adapted to patients on the basis of risk stratification [7]. This interdisciplinary management can achieve a 5-year survival rate between 50% to 90% [10,11,12,13,14,15].

However, regardless of long-term survival, treatment-related adverse effects, including neurological toxicity, hematological toxicity, polyneuropathies, endocrinopathies, and cognitive disorders remain a relevant issue, involving up to 80% of patients, thus representing a strong incitement for the search for more innovative and less impactful therapeutic strategies [9].

MB is a heterogeneous disease, both clinically and molecularly. Current MB classification, according to the fourth (2016) and fifth (2021) edition of the WHO Classification of Tumors of the Central Nervous System (CNS) [16,17], includes both histological and molecular criteria, defining four molecular types (wingless-type (WNT)-activated, Sonic Hedgehog (SHH) activated TP53-wildtype, SHH-activated and TP53-mutant, and non-WNT/non-SHH) and four histological patterns (classic, desmoplastic/nodular, MB with extensive nodularity, and large cell/anaplastic).

The diverse molecular types show significantly different age, molecular and clinical profiles [17,18,19,20,21,22,23,24,25,26,27]. WNT MB harbors deregulation of the WNT pathway, mostly caused by activating somatic mutations in the CTNNB1 gene, and exhibits the most favorable prognosis across all the subgroups, rarely being metastatic [17,18,19,20,21,22]. It is characterized by a high tendency to bleed and intralesional hemorrhage. SHH-activated MB is the most common subgroup in the adult population, accounting about 60% of adult MBs, with a 5-year overall survival (OS) rate of about 50–70% in the absence of TP53 mutation [22,23,24,25,26,27].

The clinically meaningful contribution provided by the latest editions of the WHO classification lies in having finally opened the way to the development of molecularly adapted treatment strategies for MB patients.

As a result, the landscape of clinical trials has significantly changed since the discovery of distinct molecular types, and multiple targeted agents are currently under investigation. SHH-activated adult MBs, particularly, have become a very appealing focus in research for the preliminary evidence of activity of the SHH-inhibitors vismodegib and sonidegib [28,29,30,31,32,33]. These small molecules are effective against MBs carrying SHH mutations, that in adults are at the level of SMO or upstream the SMO [34], and are free from one of the most feared side effects, the premature growth plate fusion, in the adult population.

The challenges we encounter in clinical practice suggest that there are two primary research objectives for adult MB: first, to design clinical trials specifically stratified by age and tumor biology, to develop rationally personalized treatments; second, to identify new experimental agents with improved acute and chronic toxicity profiles.

This review provides recommendations on the clinical management of adult MB, including basics on pathology, molecular biology, neuroradiology, neurosurgery, radiotherapy, chemotherapy and novel targeted therapies, as well as future perspectives.

## 2. Molecular Genetics

While histopathological MB features are nearly indistinguishable from those observed in children, the distribution of molecular types is markedly different, with SHH-activated MB representing by far the most common molecular group in adults [35].

The recently released 2021 WHO Classification of Central Nervous System (CNS) Tumours [17] classifies MB primarily according to molecular features, recognizing histopathological patterns of clinical utility and thus promoting an integrated molecular and histopathological diagnostic approach. Four main genetically defined types, including SHH-activated TP53-wildtype, SHH-activated TP53-mutant, WNT-activated and non-WNT/non-SHH MB (including group 3, 4 MB) [17,36] are identified, which allow for an accurate risk stratification (Table 1, Table 2 and Table 3).

Goschzik T et al. [37] reported a 5-year event free survival (EFS) of 60% and 5-year overall survival (OS) of 75.6%, in a cohort of 117 adult MBs. For SHH-TP53mut-MBs, EFS and OS were poor, whereas all other types had an outcome similar to published standard-risk patients with a 5-year EFS/OS between 60% and 80% each. No significant survival differences in log-rank tests were reported between WNT-MBs, SHH-TP53wt-MBs, and non-WNT/non-SHH-MBs (Table 1).

### 2.1. Medulloblastoma Histologically Defined

In absence of molecular information, MB can be diagnosed based on morphological features as MB histologically defined.

Histopathological subtypes of MB include: classic, desmoplastic/nodular (D/N), MB with extensive nodularity (MBEN), and large cell/anaplastic (LC/A).

Classic MB is a blue cell tumor without distinctive architectural features composed of cells with high NC ratio, a variable degree of nuclear anaplasia from slight to moderate and the frequent presence of Homer–Wright rosettes. Some cases show evidence of neuronal/neurocytic differentiation with the presence of nodules which, however, lack perinodular reticulin deposition and internodular desmoplasia. These features are typical of classic rather than D/N MB.

Nodular/desmoplastic MB is characterized by the presence of varying degrees of neuronal differentiation, typically with a nodular appearance confirmed by the presence of a perinodular reticulin stain. The differentiating nodules characteristically show intense synaptophysin positivity and limited proliferation/Ki67 labeling. D/N features can be focal, and MB with extensive nodularity, as described in very young children, is not seen in adults.

Large cell/anaplastic MB is characterized by severe nuclear anaplasia and/or large cell appearance, i.e., cells with large roundish nuclei and prominent nucleoli. Synaptophysin stain is diffusely positive.

Rare MB examples show evidence of melanotic or myogenic differentiation at times easily identifiable on H&E slides, sometimes exclusively by appropriate immunohistochemical markers. Mitotic activity is variable but generally high in the poorly differentiated components of the tumor. Prominent cell apoptosis and necrosis are frequently present. By immunohistochemical stains, MB cells are typically synaptophysin positive and may express to some extent GFAP, highlighting cells with scant cytoplasm, while OLIG2 stain is negative or at most only focally positive. Vimentin stain is typically negative. A markedly infiltrative pattern of growth can be seen in MB especially in adults and may make it difficult to distinguish MB from a high grade diffusely infiltrating glioma occurring in the cerebellum. In adults, especially older adults, differentiating MB from metastatic poorly differentiated/small cell carcinoma to the cerebellum is also critical. MB does not express cytokeratin and TTF1, markers typically expressed in metastatic poorly differentiated/small cell carcinoma.

Surrogate immunohistochemical markers are frequently used in clinical practice to predict molecular groups. In our experience, an immunohistochemical panel including YAP1, GAB1 and beta-catenin, simplified from the original panel described by Ellison et al., which also included filamin stain, can reliably identify in most cases MB SHH-activated (as well as distinguish TP53-wildtype and mutant MB with the addition of p53 immunostaining), WNT-activated, non-WNT/non-SHH MB.

SHH-activated MB is typically GAB1 and YAP1 positive, with a characteristic pattern of expression in the primitive/poorly differentiated components and absent stain in differentiating nodules. Beta-catenin expression is cytoplasmic. WNT-activated tumors typically show nuclear expression of beta-catenin (at least focally), expression of YAP1, but not GAB1. Lymphoid-enhancing factor 1 (LEF1), a transcription factor mediating WNT/β-catenin signaling, is overexpressed in WNT-activated MB and can further solidify the diagnosis [38]. Non-WNT non-SHH MB are negative for YAP1 and GAB1 and show cytoplasmic expression of beta-catenin. Immunostaining for p53 protein is generally sufficient to assess TP53 mutational status of most SHH-activated MB, and TP 53 mutation results in strong and diffuse expression in most tumors.

Additional markers have been proposed, including p75-NGFR and OTX2, to help with assignment to a specific MB entity and exclude other mimic histological tumors [17].

There are two major limitations of the immunohistochemical panel in “molecularly” classifying MB: (1) the inability to distinguish between groups 3 and 4, which is, however, not relevant in adults; (2) the occurrence of inconclusive immunohistochemical results, especially in rare histopathological subtypes (such as MB showing melanocytic/rhabdomyoblastic differentiation). Molecular analysis may, therefore, be critical in reaching a definitive diagnosis.

### 2.2. Medulloblastoma Molecularly Defined

The histopathological patterns have characteristic and specific associations with molecular subgroups. For instance, all morphologically defined nodular/desmoplastic MB correspond to SHH-activated MB and most WNT-activated MB have classic morphology. There is, however, no 100% correspondence between histopathology and molecular groups.

Molecular classification of MB, including WNT-activated, SHH-activated and TP53-wildtype, SHH-activated and TP53-mutant, non-WNT/non-SHH MB groups, can be achieved using a variety of molecular techniques, including methods based on mRNA such as NanoString and RNA-Seq [39,40,41,42,43], or based on DNA analysis such as copy number variations, epigenetic classification by whole-genome methylation array [44,45]. To date, whole-genome methylation profiling is considered the gold standard diagnostic method, has been obtained mainly in children while these methylation data in adults is limited [41,42,45]. Comparative studies are being added to MB clinical trials in adults to further define the optimal diagnostic approach [35].

MB molecularly defined in adults [11,24,46,47] shows a predominance of SHH-MB, typically TP53-wildtype [37], while SHH TP53-mutant MB cases seem to occur predominantly in children and adolescents. The frequency of WNT-MB is similar to that published for children (15% vs. 10%), and non-WNT/non-SHH-MB is relatively rare (2.6%) in adults compared to childhood MB (25%) [37,46,47]. The group of non-WNT/non-SHH-MBs in adults nearly consists of Group 4 tumors only [11,24,48]. Molecular groups in adult MB are summarized in Table 1 according to their mutational pattern, copy number variations and global methylation profile. Each group is characterized by distinct clinicopathological and molecular features.

### 2.3. Medulloblastoma SHH-Activated and TP53-Wild Type

SHH-activated TP53-wildtype MB represents the most frequent type [11], accounting for approximately 60% of MB in adults [37]. While SHH activation is, predominantly, associated with pathogenic mutations of Smoothened (SMO) in adults, Patched (PTCH1) mutations can be found in all age groups.

Other frequent genetic alterations have been reported, including ELP11, DDX3X, and KMT2D mutations. PPM1D amplification has also been described as a driving molecular event in adult SHH-activated TP53-wildtype MB [49]. Copy number variations. involving 9q loss and 9p gain, are the most frequent abnormalities observed in this group [17]. The majority of SHH-MB TP53-wildtype tumors show desmoplastic/nodular histological patterns, which may also be present in focal areas only, while MB with extensive nodularity is not observed in adults. Less frequently, SHH-MB TP53-wildtype has classic histology.

### 2.4. Medulloblastoma SHH-Activated TP53-Mutant

TP53 mutation is rare (2.6%) in adult SHH-MB [37], but may occur de novo in recurrent tumors. TP53 gene sequencing is recommended in all SHH-MB by the World Health Organization [17].

Most instances of SHH-MB TP53-mutant in adults show somatic TERT promoter, DDX3X and U1 snRNA mutations [34].

Amplification of MYCN and GLI2 has been reported. In adults, MYCN amplification and TP53 mutation tend to occur together and are associated with a poor prognosis [17].

Loss of several chromosomes such as 17p, 3p, 10q, 14 q and gain of 3q has been reported in SHH-activated TP53-mutant MB. DNA methylation profile aligned with a SHH-activated MB, either TP53-wildtype or TP53 mutant, is considered the gold standard method for determining MB group status in children. Four molecular subgroups of SHH-activated MB are emerging [23,37,44,50,51,52,53] (Table 1).

A consensus regarding these SHH subgroups and their defining features has not yet been reached through an international cooperative meta-analysis. Of the four proposed subgroups, the main subgroup occurring in adults is SHH-4, which is associated with near-universal U1 and TERT mutations and frequent somatic PTCH1 or SMO alterations [54].

TERT promoter mutations have been reported in most adult MBs, but only rarely in childhood MB [34,46,55].

SHH-3 is the other subgroup that may arise in adult patients and is associated with TP53 and ELP1 mutations [54]. The last two molecular SHH-subgroups occur mainly in young children: one (SHH-1) is enriched with somatic and germline suppressor of fused (SUFU) mutations and chromosome 2 gain, and the other (SHH-2) is characterized by 9q loss and DNMB morphology.

### 2.5. Medulloblastoma Non-WNT/Non-SHH-MB

Non-WNT/non-SHH-MB represent 22.2% of adult MBs and are classified as group 3 and group 4 [37]. Group 3 is exceedingly rare in adults and the majority of adult Non-WNT/non-SHH-MB cases belong to subgroup 4 [11,21,24]. The most frequent genetic alterations in groups 3 and 4 involve KDM6A, OTX2, ZMYM3, KMT2D, TBR1 and PRDM6. Aberrant overexpression of PRDM6 is specific to group 4 MB. Cytogenetically, isochromosome 17q is present in most cases. In addition, some cases show amplifications such as CDK6, while amplifications of MYC or MYCN are rare in adults.

DNA methylation profiles of non-WNT/non-SHH-MB align with group 3 and group 4 MB, among which eight molecular subgroups have recently been identified and classified in a spectrum of group 3/group 4 MBs by DNA methylation profiling [23,37,50] (Table 1). Based on analysis of DNA methylation and transcriptomic data, these molecularly heterogeneous subgroups among group 3 and group 4 MBs were identified with distinct clinical and genetic associations [56].

In an international meta-analysis using DNA methylation and transcriptome data, Sharma et al. [57] found eight robust subgroups within MB group 3/4. Group 3 MBs consist only of subgroups 2, 3, and 4, whereas group 4 MBs include uppermost subgroups 6, 7, and 8. Subgroups 1 and 5 are intermediate subgroups, showing the molecular and cellular features of both group 3 and group 4 MBs [56]. Classic morphology is observed in most non-WNT/non-SHH MBs, but subgroup 2 are more frequent large cell/anaplastic tumors. Metastatic disease at presentation is relatively frequent in subgroups 2–5. A rather poor outcome is associated with tumors in subgroups 2 and 3 where MYC/MYCN amplification are identified.

### 2.6. Medulloblastoma WNT-Activated

MB WNT-activated is the least common of the molecular groups in adults. In a recent study, 14.5% of adult MBs show WNT activation, mostly caused by activating somatic mutations in the CTNNB1 gene [37]. For a definite diagnosis of WNT-activated MBs sequencing is recommended to confirm the presence of a CTNNB1 mutation. Inactivating mutations in APC genes are rare and might suggest germline mutations (familial polyposis coli) [17]. As both TERT and IDH1 mutations are genetic hallmark events in subgroups of gliomas, their molecular presence alone, without integration with the histological and immunohistochemical data, does not permit the differential diagnosis between malignant glioma and MB in cerebellum.

Monosomy of 6 is found in >80% of WNT-activated MB, cytogenetically characterizing this group, especially in children [17]. The DNA methylation profile aligned with WNT-activated MB. Two molecular subgroups of WNT-activated MB have been proposed, WNT alfa and WNT beta [17]. WNT alfa MB is characterized by a monosomy of 6 and arises in children. WNT beta MB is diploid for chromosome 6 and arises in older children and young adolescents. The majority of WNT-MB shows classic histology.

## 3. Diagnostic Imaging

MB patients typically present with symptoms of increased intracranial pressure based on an obstructive hydrocephalus and/or cerebellar symptoms (e.g., ataxia, gait disturbance) due to the tumor’s location in the posterior fossa. Thus, in the acute clinical setting, the first imaging technique is routinely an unenhanced computed tomography (CT) to rule out or confirm a potential brain lesion. Herein, MBs usually present as a hyperdense mass in the posterior fossa due to high tumor cellularity [58]. To further characterize the intracranial lesion, usually a magnetic resonance imaging (MRI) examination is conducted, which is the modality of choice for diagnostic imaging and radiological follow-up examinations [59].

The basic brain MRI protocol for diagnostic image acquisition and response assessment in MBs comprises of a fluid attenuated inversion recovery (FLAIR) MR sequence (slice thickness ≤ 4 mm), a T2-weighted MR sequence (slice thickness ≤ 4 mm), a diffusion-weighted (DWI) MR sequence (slice thickness ≤ 4 mm) as well as isovoxel 3D pre- and post-contrast T1-weighted MR sequences (slice thickness = 1 mm). To avoid vascular structures obscuring or mimicking leptomeningeal brain metastases, 3D post-contrast T1-weighted fast spin echo sequences are recommended. Moreover, FLAIR images should be obtained after the intravenous application of gadolinium-based contrast agents because of its high sensitivity to detect leptomeningeal metastases [60].

**Figure 1:**
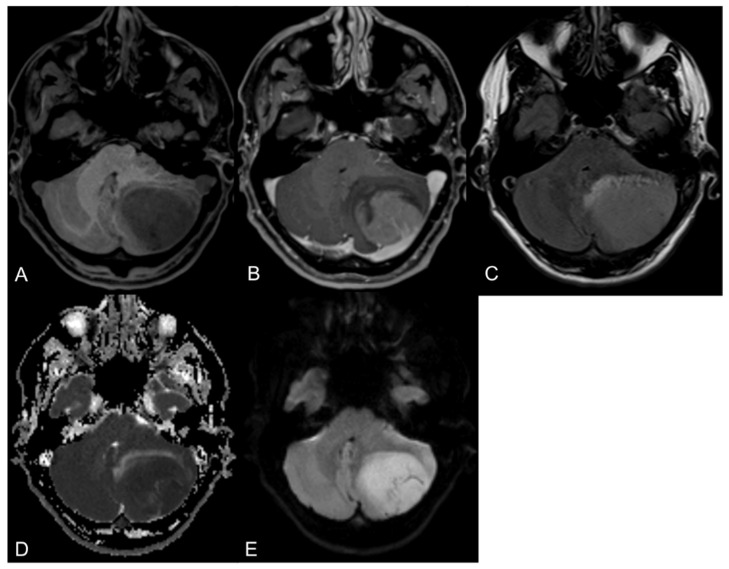
Axial MR images of a 46-year-old male patient with a medulloblastoma located in the left cerebellar hemisphere. The tumor shows a characteristic hypointense signal on T1-weighted images (**A**) with a mainly homogenous contrast enhancement (**B**), a hyperintense signal on FLAIR images (**C**) and a considerable diffusion restriction (**D**,**E**).

In MR imaging, MBs are characterized as intra-axial tumorous lesions with sharp margins and little edema. On T2-weighted images, lesions are hypo-to-hyperintense, and on T1-weighted images, lesions are typically hypointense in comparison to the normal grey matter depending on the histopathological and molecular subtype. After the intravenous application of gadolinium-based contrast agents, the lesions show moderate to strong contrast. Due to the hypercellular nature of this brain tumor entity, it is recommended to routinely obtain diffusion-weighted images, on which MBs present with restricted diffusion [60]. In Figure 1, MR images of a MB (SHH-mutated) in the left cerebellar hemisphere are presented.

In contrast to MB in children, which are located in the cerebellar midline arising from the vermis, MBs in adult patients are predominantly represented by the sonic hedgehog (SHH)-mutated tumor subtype (60–70%), which is primarily located in the cerebellar hemispheres [11]. This molecular subtype is characterized by a strong diffusion restriction and an increased perifocal edema. In contrast the wingless (WNT)-activated subgroup of MB in adult patients (20%) shows a lower tumor volume at diagnosis and reduced subependymal macrometastasis in comparison to the other molecular tumor subtypes [11,61].

For further therapy monitoring, especially in clinical trials, the RAPNO committee developed specific response assessment criteria for MB and other seeding tumors [60].

Complete response (CR) is defined as the complete absence of all tumorous lesions in the brain and spine, independent if they are measurable or unmeasurable, or if they show contrast enhancement or not for at least 4 weeks.

The definition of partial response (PR) is determined as a ≥50% decrease (compared with the baseline examination) in the sum of the product of perpendicular diameters of all (≤4) measurable lesions for at least 4 weeks and stable or reduced non-measurable disease.

Disease progression (PD) is defined as a ≥25% increase in the sum of the products of perpendicular diameters of all (≤4) measurable lesions (compared to the smallest measurement at any time point), significant progression of non-measurable disease not assigned to prior therapy, or new tumoral lesions (all lesions suspected to be treatment related should be confirmed by biopsy). If none of the above criteria are met, it is defined as a stable disease (SD).

Postoperative MR imaging to detect residual tumor formations is ideally obtained within 48 h after surgery. In case of substantial postsurgical changes that may mask the presence of tumor remnants, it is recommended to repeat MRI 2–3 weeks after surgery [60].

MBs have a tendency for leptomeningeal dissemination in the brain, but also seeding metastases along the spinal axis. Therefore, diagnostic MRI should also include the entire spine. Spinal MRI examinations should ideally comprise of a sagittal pre- and post-contrast T1-weighted sequence (slice thickness ≤ 3 mm), an axial post-contrast 3D T1-weighted sequence gradient echo sequence (slice thickness ≤ 4 mm) and a 3D myelographic T2-weighted sequence (slice thickness ≤ 1 mm) particularly highlighting small spinal leptomeningeal metastases that are difficult to detect on T1-weighted images [62]. Preoperative screening for spinal leptomeningeal metastases is preferable whenever feasible, avoiding postoperative changes that obscure spinal leptomeningeal dissemination [60]. However, there is still no international consensus on when follow-up spinal MRI examinations should be performed based on large study cohorts. Thus, so far, the RAPNO MB committee recommends the performance of spinal MRI examinations along with brain MR imaging in all patients with positive CNS cytology or preexisting leptomeningeal dissemination at baseline examination and in MB patients who are experiencing new spinal symptoms [60].

If patients present symptoms of extra-CNS involvement, systemic staging (such as contrast enhanced CT scan of the chest, the abdomen and the pelvis; whole body PET CT; bone scan, etc.) is required [59,63].

Given the high costs and the unavailability of molecular testing at various institutions around the world, there is a high need of imaging-based genetic tumor type correlation. Thus, currently various research groups are focusing on, amongst other topics, the MRI-based distinction of molecular MB subtypes using artificial intelligence. Recently, a large retrospective multicenter study established MRI-based markers that correlated with molecular genomic sequencing of MBs [64]. Using deep-learning algorithms, they could, e.g., uncover therapeutically relevant, low-risk WNT MB as well as high-risk childhood SHH MB with a high reliability. However, these clinical trials are predominantly based on pediatric MB patients and have a retrospective study design, thus, the results of these studies should be validated for the adult MB patient population in prospective clinical studies in the future.

## 4. Neurosurgical Aspects in Adult Medulloblastoma Surgery

### 4.1. Preoperative Management

After brain and spine magnetic resonance imaging is obtained, each patient should be discussed in a multidisciplinary brain tumor board. A chest and abdominal CT should also be considered to exclude a malignancy. The different treatment options should be discussed in detail with a special emphasis on age, co-morbidities and hence surgical risk. While in the pediatric population there is always a strategy to an attempt at GTR, in an elderly population the option of a biopsy should be discussed, if the surgical risk is deemed too high.

However, whenever safely possible, a surgical resection should be attempted. Goal of the surgery is to provide tissue for histopathological and molecular examination, reduction of intracranial pressure by treating a potential hydrocephalus and reduction of the tumor mass effect. Administration of corticosteroids prior to surgery, typically with Dexamethasone 8 mg once daily in the morning [65], can help prepare for surgery through reduction of vasogenic tumor oedema. Primary antiepileptic prophylaxis is not indicated in patients without seizures. Despite mainly being a tumor of the posterior fossa, a patient could suffer a seizure because of hydrocephalus. In that rare event, patients should receive anticonvulsant drugs preoperatively.

### 4.2. Surgical Considerations

Medulloblastoma frequently presents emergently with signs and symptoms of increased intracranial pressure due to obstructive hydrocephalus. The presence of moderate to severe hydrocephalus has been demonstrated in 30–40% [66,67,68], thus not rarely requiring an emergent pre- or intraoperative CSF diversion. An emergent external ventricular drain through Kochers point or through a Frazier burr hole can temporarily be placed. An alternative procedure is an endoscopic third ventriculostomy (ETV). In most cases, surgery with definite tumor removal relieves the obstruction-causing hydrocephalus and no further CSF diversion procedure is needed [69]. Ventriculo-peritoneal shunting prior to tumor resection is not recommended due to the risk of upward herniation and of cell seeding into the peritoneal cavity.

The surgical plan should always be an attempt at GTR with a residual volume of less than 1·5 cm^2^. This is, however, not always safe or feasible. In such cases, maximal safe resection leaving a residual tumor behind rather than causing neurological deficits by aggressive resection is recommended [70]. In addition, in case of a disseminated disease, tumor debulking is indicated to relieve symptoms from mass effect.

In contrast to the pediatric population, up to 70% of adult medulloblastomas occur in cerebellar hemispheres with a predominance of the SHH subgroup [71]. In the pediatric population, 90% of medulloblastomas occur in the midline [72,73]. Therefore, the surgical approach is more often paramedian in the adults and a safe GTR is more feasible. Another rare, but characteristic location for adult population is cerebellopontine angle requiring a retrosigmoidal approach [74]. The suboccipital approach for midline tumors is performed in a prone position. The paramedian for cerebellar hemisphere tumors can be done in a prone or lateral position depending on the surgeon’s preference. The retrosigmoid approach can be done in a lateral or supine position with elevated ipsilateral shoulder. For all approaches, the head is fixed in a head clamp with pins. We always recommend using neuronavigation and an ultrasonic tissue aspirator. Intraoperative neuro-monitoring is used for the rare cases of cerebellopontine angle location, but is not mandatory for cerebellar lesions. A gross total resection was achieved in 60–90% of adult medulloblastoma series [66,67,68]. There are no data to support the use of fluorescence agents such as 5-ALA in adult medulloblastoma surgery. In the pediatric population, 5-ALA fluorescence was found to be present only in 22% of patients [75]. Intraoperative MRI may be used to assess the extent of resection. Nevertheless, a high-quality post-operative MRI within 48 h is necessary for appropriate staging. A second look surgery should be considered if a residual tumor of more than 1.5 cm^2^ is noted on the postoperative scan.

Surgical complications occur up to 30% patients and include: hematoma, cerebrospinal fluid fistula, infection/meningitis, hydrocephalus, pseudomeningocele, and cranial nerve palsy [68]. Postoperative hydrocephalus, requiring permanent cerebrospinal fluid diversions, occurs in 14–37.5% of patients [68,71]. Cerebellar mutism has been rarely described in adult patients after medulloblastoma surgery and usually resolves within 30 days [76,77].

Intraoperative CSF sampling can be obtained in order to assess CSF seeding. However, standard of care is CSF sampling via lumbar puncture, ideally prior to or 2 weeks after surgery [78]. Surgery should take place in high-volume centers (100 brain tumors per year).

## 5. Role of Chemotherapy and Radiotherapy

Within the mid of the 20th century the combination of tumor resection and adjuvant craniospinal irradiation (CSI) became available as the first curative treatment in children with MB [79]. With this concept overall 5- and 10-year survival rates of 56% and 43% were achievable [80]. Later, the addition of adjuvant chemotherapy with CCNU, Cisplatin and Vincristin lead to 5-year survival rates of >80% [81,82] and established the backbone of MB treatment. In the intent to decrease neurotoxicity, reduced-dose CSI with 23.4 Gy was applied in large clinical trials together with chemotherapy. The 5-year PFS of this approach in standard risk patients was around 80% [83,84].

More intensive approaches with hyperfractionated radiotherapy did not lead to a superior survival compared to conventional radiotherapy in average risk MB [85]. Together, these studies set the current standard for average risk MB in childhood involving 23.4 Gy CSI with single doses of 1.8 Gy, combined with CCNU, Cisplatin and Vincristine. The question of radiotherapy dose reduction has not been investigated in a randomized way in adults; however, single-arm data indicate that a dose reduction to 23.4 Gy is feasible without losing efficacy [86].

### 5.1. Why It Is Difficult Translating Pediatric Experiences in the Adult Counterpart

While treatment has evolved gradually in children mostly based on the results of large randomized clinical trials, this was not the case for adult patients [7]. Treatment of adults has therefore been more or less extrapolated from experiences in children. However, translation of pediatric experience to adult counterparts has severe limitations due to several reasons: mainly, molecular differences and toxicity differences between children and adults.

#### 5.1.1. Molecular Differences between Tumors

Adult and pediatric MBs are biologically and prognostically distinct [11,23]. SHH-MB (70%), Group 4 (20%) and WNT subtype are the dominant genetically defined subgroups. Adult SHH-MB differ from pediatric SHH tumors in a low frequency of p53 mutations and a higher frequency of TERT promoter mutations (SHH Delta) [51]. Other examples of distinctions of adult and pediatric MB are the worse prognosis of group 4 and WNT tumors, and the presence of WNT beta instead of a WNT alpha subgroup [51]. Generally, adults with MB bear a worse prognosis and show a tendency to also relapse later in their disease course [87].

#### 5.1.2. Differences between Toxicity in Children and Adults

Treatment toxicities differ significantly between children and adults. Chemotherapy-related hematologic toxicity and peripheral neurotoxicity appear more prominent in teenagers and young adults [88,89], while central neurotoxicity is more pronounced in pediatric patients, with increasing detrimental effects of radiotherapy with decreasing patient age [90,91,92]. Further, young children appear more prone to growth impairment due to radiotherapy [93]. With consideration of limited clinical trial data, long-term detrimental effects like neurotoxicity, ototoxicity or endocrinopathies are much less studied in adults and require further prospective examination.

### 5.2. Results of German and Italian Trials for Adult MB

Chemotherapy treatment recommendations for adults are based on sub-cohorts of pediatric trials and single arm prospective trials. The Packer regimen (vincristin during radiotherapy, followed by lomustine, cisplatin and vincristin) [82,83] was established in children [10,85,94] and has also been used in adults. In a sub-cohort of the pediatric HIT-2000 trial, 49 non-metastatic adults received combined radio-chemotherapy and experienced a 4-year event-free survival of 74% and OS of 94% [10].

In an Italian phase II trial, 26 high-risk patients received two cycles of upfront chemotherapy followed by radiotherapy and chemotherapy. The 5-year PFS rate was 69%, and the 5-year OS rate was 73%. A follow-up analysis on low-risk patients from the same trial showed that cisplatin-based chemotherapy after radiotherapy obtained a 10-year OS of 100% compared to 78.6% in patients treated with radiotherapy alone (*p* = 0.079) [15,95]. The prospective German phase II NOA-07 trial used the Packer regimen. The regimen was feasible for the majority of patients for up to 6 cycles, but leukopenia, polyneuropathy and ototoxicity were major toxicities. The 3-year EFS and OS rates were 66.6% were and 70.0% [9].

Meta-analyses also suggest an increased efficacy of radio-chemotherapy in comparison to radiotherapy. In a large meta-analysis, adult patients receiving chemotherapy survived significantly longer (mOS: 108 months, 95% CI 68.6–148.4) than those treated with RT alone (mOS: 57 months, 95% CI 39.6–74.4) [14]. A retrospective analysis of the National Cancer Data Base registry also confirmed the superiority of postoperative radiotherapy and chemotherapy over radiotherapy alone [96].

### 5.3. New Techniques of Radiotherapy

Craniospinal radiotherapy is a potentially toxic treatment. Within the last few decades, advanced 3D conformal treatment techniques like volumetric arc therapy (VMAT) and tomotherapy were introduced in order to reduce the dose to organs at risk outside the target volume and enhance speed and quality of treatment. However, these techniques share the disadvantage of increasing low dose irradiation volumes in the body [97,98,99,100] with a potential persisting risk for secondary malignancies in other organs.

### 5.4. Proton Treatment

Tumor control does not differ between proton and photon treatment [101,102], but due to different physical properties of protons, normal tissue outside the target volume can be better spared from irradiation dose significantly [103,104,105,106,107].

This implies several advantages of proton radiotherapy. Most importantly, early (e.g., hematotoxicity [108,109]) and late toxicity (e.g., endocrine dysfunction, cardiotoxicity and ototoxicity) appear to be reduced with proton treatment [109,110,111,112,113]. As a further advantage, the risk of secondary cancers in organs outside the target volume can be potentially diminished [114,115,116,117,118,119,120].

However, apart from estimations from plan comparisons, clinical evidence concerning this field is still sparse. In a retrospective series of 115 children with MB after photon CSI or proton CSI, the secondary tumor incidence rates were not different between the two groups [121]. Prospective data from controlled clinical trials on toxicity and secondary cancers are sparse but needed in adults with MB.

### 5.5. EORTC 1634-BTG Trial/Alliance AMBUSH Trial

Two prospective randomized trials have recently been initiated.

The Europe-based EORTC 1634-BTG/NOA-23 trial randomizes patients between standard dose (35.2 Gy, followed by a boost of 19.8 Gy to primary sites) vs. reduced-dosed cranio-spinal radiotherapy (23.4 Gy, followed by a boost of 30.6 Gy to primary sites) and SHH-subgroup patients between the SMO-inhibitor sonidegib in addition to standard radio-chemotherapy with vincristin, lomustin and cisplatin vs. standard radio-chemotherapy alone to improve outcomes in view of decreased radiotherapy-related toxicity and increased efficacy. Tumor tissue, blood and cerebrospinal fluid, magnetic resonance imaging, radiotherapy quality, HR-quality of life, cognition, fertility and long-term outcomes will be monitored in this trial [8].

The US-based AMBUSH trial assigns patients 18 years of age or older with MB to an average risk non-SHH subgroup, an average risk SHH-subgroup and a high-risk subgroup. All patients will receive protocol-directed comprehensive treatment with 23.4 Gy radiotherapy on the neuroaxis followed by a boost of 36 Gy to primary and metastatic sites and chemotherapy with cisplatin, vincristine and cyclophosphamide. Patients within the average risk SHH-subgroup will be randomized to a smoothened inhibitor or placebo as maintenance therapy for one year [35].

### 5.6. New Drugs

Recent advances in the molecular characterization of MB have led to the development of targeted therapies, which have a better toxicity profile and a more selective spectrum of efficacy than the traditional cytotoxic therapies.

Among the described molecular subgroups, the SHH-activated, TP53 wild-type subgroup is highly overrepresented in adult MB, with around 60 to 70% of cases [11], representing the population of choice for the development of targeted agents acting by the inhibition of the SHH-signaling pathway. SHH is a signaling cascade playing a crucial role in cellular proliferation, tissue morphogenic defects and tumor growing [34,122,123,124,125], and is constitutively over-activated by driver mutations in PTCH1 or SMO and by mutations downstream of SMO, involving SUFU, a negative regulator of SHH signaling, and GLI proteins.

Vismodegib and sonidegib, two highly selective SHH inhibitors targeting the SMO receptor, have demonstrated a favorable toxicity profile and an encouraging efficacy in preclinical and clinical trials of patients affected by MB with activation of the SHH pathway [28,29,30,31,32,33,126,127,128,129].

A major issue for SHH-inhibitors in MB patients is the rise of early drug resistance, mostly associated with genetic alterations within or downstream the SMO, among SUFU and GLI, as well as among Phosphoinositide-3 kinase (PI3K) and MEK pathways [130,131,132]. Notably, for the future treatments of SHH-activated tumors, the use of combination therapies with SMO inhibitors and PI3K/MEK inhibitors as well as SMO inhibitor plus temozolomide [33] or SMO inhibitors in addition to standard radio-chemotherapy [8] has been proposed, suggesting that concomitant treatment might increase the therapeutic benefits of these small molecules SMO antagonists.

## 6. Importance of Centralizing Adult Medulloblastoma Patients in Hub Centers

Since adult MBs are rare and heterogeneous, it is difficult for clinicians to gain sufficient experience in the field.

Rarity leads to major problems: difficulties in achieving correct diagnosis and treatment in adequate times; difficulties to identify specialists and centers of reference; difficulties in conducting clinical studies with an adequate sample; limited interest in research by pharmaceutical companies due to the reduced economic feedback; logistical and economic difficulties for patients given the scarcity of referral centers.

Inequalities in European countries exist both in the access to diagnosis and treatment, as well as in the access to information.

This is even more likely if low numbers of cases are treated/year, or if patients are handled without a multidisciplinary approach. It is, therefore, strongly recommended that the treatment of MB take place at designated centers of reference.

The centralization of care in centers with expertise in the treatment of MB has been shown to improve the overall quality of care and survival of MB patients [133], providing more accurate diagnosis, more timely treatment through multidisciplinary teams, and the possibility of enrollment in clinical trials.

It is recommended that patients with MB are followed by a multidisciplinary team composed of a neurosurgeon, pathologist, neuroradiologist, medical oncologist, radiotherapist, neurologist, psychologist, and a specialized palliative care team, to provide an approach capable of satisfying all the patient’s needs during the various stages of the disease.

The presence of a multi-disciplinary group of specialists is considered an indispensable criterion for the center inclusion into the ERN-European Reference Network. The ERN reference network for rare diseases and cancers was born in 2017 as a European organization for the improvement and enhancement of specialized care for patients with rare and complex diseases and rare cancers. These networks aspire to create stable collaborations aimed at sharing knowledge and coordinating health care between centers of excellence for the care of patients with rare diseases and rare cancers. In addition, ERNs were also initiated to support scientific research, define guidelines and promote educational activities for rare diseases and cancers.

In order to enter the ERNs, the centers have to meet some basic requirements, first of all ensuring the presence of a multidisciplinary team completely dedicated to the disease.

Out of a total of 24 ERNs, three are specifically dedicated to rare tumors: EURACAN (rare solid tumors of adults, including brain tumors), EuroBloodNet (hematological diseases and rare hematological tumors) and PaedCan (rare pediatric tumors). Within EURACAN, domain 10 is dedicated to brain tumors.

## 7. Conclusions and Future Directions

MB is a very rare tumor in the post-pubertal and adult population, with a distinctly unique profile from a clinical, molecular, prognostic and therapeutic point of view.

Despite the relevant innovations in the pathology and molecular biology field emerging from the recent 2021 WHO classification for the MB diagnosis, practice-changing clinical trials modifying medical treatments and radiotherapy options, especially in adult MB, are still lacking.

Irrespective of the individual risk and of the molecular subgroup, the standard of care remains surgery followed by craniospinal irradiation and multi-agent chemotherapy, with a wide spectrum of short and long-term toxicities of various types, such as hematological, neurocognitive and endocrinological. A large proportion of patients experience significant worsening of the quality of life and impairment of the physical and psycho-social sphere, therefore, the development of novel treatment strategies minimizing the toxicities remains an area of fervent research [9].

Lessons from the use of personalized targeted therapies in oncology have shown the advantage of considering the molecular profile as the primary criteria for the clinical trial design. Future research dedicated to adult MB should move forward in the direction of personalized treatments, stratifying patients by age and molecular profile.

This will require consistent academic and financial investments from third-party funders as well as the pharmaceutical industry, research institutes and scientific societies.

## Figures and Tables

**Table 1:** Medulloblastoma molecular types.

Genetically Defined	WNT-Activated	SHH-Activated	Non-WNT/Non-SHH (Group 3 & 4)											
		TP53-Wildtype	TP53-Mutant											
Frequency in Adults (%) **	14.5	60.7	2.6				22.2							
**Histologically** Defined *	Classic	Desmoplastic/ nodular	Large cell/anaplastic				Classic, Large cell/anaplastic							
**Immunophenotype**	Cytoplasmic & Nuclear Beta catenin YAP1 positive GAB1 negative	Cytoplasmic Beta catenin YAP1 positive GAB1 positive p53 low expression/negative	Cytoplasmic Beta catenin YAP1 positive GAB1 positive p53 high expression				Cytoplasmic Beta catenin YAP1 negative GAB1 negative							
Metastasis in adults **	M0 83.3% M1–M3 16.7%	M0 92% M1–M3 8%?	M0 100% M1–M3 0%				M0 30% M1–M3 70%							
Subgroups ***		SHH-1	SHH-2	SHH-3	SHH-4	SHH-3 TP53-mutant	1	2	3	4	5	6	7	8
		SHH-I SHH-ß SHH-infant	SHH-II SHH-ƴ SHH-infant	SHH-α SHH-child	SHH-δ SHH-adult	SHH-α SHH-child								
**Frequency (%)**		15–20	15–20	20–25	30–35	10–15	3–5	10	10	8	8	8	15	25
**Cytogenetics** **FISH, MIP, Methylation Array**	Monosomy 6	2+	9q− 10q−	9p+ 9q−	3q+ 9q− 10q− 14q−	3q+ 17p− 3p− 10q− 14q	Balanced	8+ 10q+ i17q	7+ i17q 10q− 16q−	14+ 7+ 8− 10− 11− 16−	7+ i17q 16q	7+ i17q 8− 11−	7+ i17q 8−	i17q
**Driver Events Sanger-Sequencing, Pyrosequencing NGS Panel**	CTNNB1 DDX3X APC	KMT2D	PTCH1 SUFU SMO	PTCH1 ELP1 DDX3X KMT2D PPM1D	U1 snRNA TERT PTCH1 DDX3X SMO CREBBP GSE1 FBXW7	TP53 DDX3X U1 snRNA TERT MYC GLI2	GFI/ GFI1B activation OTX2 amplification	MYC, amplification GFI/ GFI1B Activation KBTBD4, SMARCA4 CTDNEP1, KMT2D mutation	MYC MYCN amplification	Not known	MYC, MYCN amplification	PRDM6 activation MYCN amplification	KBTBD4 mutation	PRDM6 Activation KDM6A ZMYM3 KMT2C mutation

* Medulloblastoma, not otherwise specified (MB, NOS): this designation indicates that the necessary diagnostic information (histological or molecular) to classify the MB according to WHO criteria are not available. Among possible scenarios, the molecular workup necessary to assign to a specific molecularly defined diagnosis could not be performed or failed, the nature of the biopsy prevents classification of the tumor even into a histopathologically defined subtype. ** Goschzik T et al. [37]. *** Data of novel molecular MB subgroups are based prevalently on the children’s population.

**Table 2:** Expected OS for molecular subtypes of adult medulloblastoma.

Molecular Subtype	5 Years Expected Overall Survival
WNT	100%
SHH TP53 MUTATED	<50%
SHH TP53 WILD TYPE	76%
NON SHH-NON WNT	47–50%

**Table 3:** Expected OS for molecular subtypes of children medulloblastoma.

Molecular Subtype	Expected Survival
WNT	>90%
SHH	
- metastatic TP53 wild type	50–75%
- non metastatic TP53 wild type	75–90%
- metastatic TP53 mutated	<50%
GROUP 3	
- metastatic	<50%
- non metastatic	75–90%
GROUP 4	
- metastatic	50–75%
- non metastatic	>90%
